# ﻿A new species of *Nitokra* Boeck, 1865 (Copepoda, Harpacticoida, Ameiridae) from the Caribbean coast of Colombia

**DOI:** 10.3897/zookeys.1128.86210

**Published:** 2022-11-08

**Authors:** Juan M. Fuentes-Reinés, Eduardo Suárez-Morales, Marcelo Silva-Briano

**Affiliations:** 1 Universidad del Magdalena, Grupo de Investigación en Biodiversidad y Ecología Aplicada, A.A 731 Santa Marta, Magdalena, Colombia Universidad del Magdalena Santa Marta Colombia; 2 El Colegio de la Frontera Sur (ECOSUR), A.P. 424, 77014 Chetumal, Quintana Roo, Mexico El Colegio de la Frontera Sur (ECOSUR) Chetumal Mexico; 3 Departamento de Biología, Universidad Autónoma de Aguascalientes (UAA), 20131 Aguascalientes, Mexico Universidad Autónoma de Aguascalientes Aguascalientes Mexico

**Keywords:** Benthic copepods, brackish waters, harpacticoids, new species, northern Colombia, taxonomy

## Abstract

Biological samples obtained from a coastal system of northern Colombia yielded male and female specimens of an undescribed harpacticoid copepod of the diverse ameirid genus *Nitokra* Boeck, 1865. The new species is a member of the genus group III. We describe the new species based on adult male and female individuals. *Nitokrapuebloviejensis***sp. nov.**, appears to be most closely related to *N.vietnamensis* Tran & Chang, 2012, but they can be separated by the following characters: 1) number of setal elements on second segment of mandibular palp, 2) P1ENP/EXP ratio, 3) relative lengths of P2, P3ENP/EXP, 4) number of elements on male P5EXP and ENP, and 5) segmentation of male antennule. In addition, *N.puebloviejensis***sp. nov.** can be confused with two other congeners: *N.taylori* Gómez, Carrasco & Morales-Serna, 2012 from South Africa and Colombia and *N.kastjanensis* Kornev & Chertoprud, 2008 from the White Sea, but the new species can be distinguished from them by: 1) number of setae on the maxillule coxa, 2) P1ENP/EXP ratio, 3) P2,P3ENP/EXP ratio, 4) female and male P5 setophore, 5) setation pattern of female P5EXP and ENP, 6) structure of female P6, 7) ornamentation of female anal operculum, 8) number of setae on male P5EXP, and 9) the male antennule segmentation. Most importantly, the presence of a group of five short setae on the medial surface of the maxilliped syncoxa allows the new species to be readily distinguished from its congeners. Only two subspecies and one species of this genus have been hitherto recorded from Colombia. A key to the 23 known American species of *Nitokra* is provided.

## ﻿Introduction

The family Ameiridae Monard, 1927 is one of the most diverse of the copepod order Harpacticoida, and it has been divided into two subfamilies: Stenocopiinae Lang, 1944, and Ameirinae Boeck, 1865, the latter being the most diverse. Currently, the family comprises 49 genera and about 303 species (Walter and Boxshall 2022). Among ameirins, *Nitokra* Boeck, 1865 is the largest genus, with 80 described species and subspecies (Karanovic et al. 2015), 23 of them recorded in the Americas. Only *N.bisetosa* Mielke, 1993, *N.taylori* Gómez, Carrasco & Morales-Serna, 2012, *N.affinisaffinis* Gurney, 1927, *N.lacustrisrichardi*Karanovic et al., 2015, *N.affiniscolombiana* Reid, 1988, *N.minorminor* Willey, 1930, *N.lacustrislacustris* (Schmankevitch, 1875) and *N.lacustrissinoi* Marcus & Por, 1961 have been hitherto recorded from the Caribbean coasts (Chappuis 1933; Suárez-Morales et al. 1996, 2006; Suárez-Morales and Reid 1998; Fuentes-Reinés and Suárez-Morales 2014a, b; Karanovic et al. 2015). *Nitokralaingensis*, included in Suárez-Morales et al. (2006) list of Caribbean harpacticoids, was not considered in this paper because it could be an undescribed species. Members of the genus occur mainly in marine environments with a wide depth range (Hendrickx and Fiers 2010), but several species have been recorded from fresh- and brackish-water habitats (Suárez-Morales et al. 1996; Karanovic and Pesce 2002), a wide range of sediment types, and some are symbiotically associated with invertebrates including flatworms, isopods, and decapods (Boxshall and Halsey 2004).

The knowledge on the diversity of *Nitokra* in Colombia is still scarce. Hitherto, only one species and three subspecies have been recorded in the country: *N.lacustriscolombianus* from Bahía Solano, Choco, *N.l.sinoi* from Ciénaga Grande de Santa Marta, Magdalena and Laguna Navío Quebrado, la Guajira, and *N.affiniscolombiensis* and *N.taylori* from Laguna Navío Quebrado, La Guajira (Reid 1988; Fuentes-Reinés and Suárez-Morales 2014a, b).

The Ciénaga Grande de Santa Marta, a large costal system of northern Colombia, was biologically surveyed during 2017 as part of an ongoing effort aiming to increase our knowledge of the Colombian aquatic biodiversity. The samples obtained yielded male and female specimens of an undescribed species of *Nitokra*. The new species is described and compared it with its closest congeners. A key to the 23 species of *Nitokra* known to occur in the Americas is also provided.

## ﻿Materials and methods

Biological samples were obtained monthly from littoral habitats of the Ciénaga Grande de Santa Marta, northern Colombia (10°52'11.25"N, 74°19'31.64"W) in July, 2022; samples were collected manually from areas with mangrove vegetation using a 25 L bucket. Water salinity, pH, and temperature were measured *in situ* with a WTW350i Multimeter.

Samples were filtered with a plankton net (45 μm mesh size) and then fixed and preserved in 70% ethanol. Copepods were sorted from the original samples and then processed for taxonomical identification, including dissection and mounting of taxonomically relevant appendages. Dissected specimens were mounted in glycerin and sealed with Canada balsam. Drawings of the mounted appendages were prepared with a camera lucida; they were also photographed using a Kodak Easy Share C140 digital camera adapted to a compound microscope. Two adult male individuals were prepared for SEM examination with a JEOL LV 5900 microscope at the University of Aguascalientes (**UAA**), Mexico; one female individual was prepared for SEM examination with a JSM-6010LA microscope at El Colegio de la Frontera Sur, Chetumal, Mexico. The whole specimens were measured in lateral position, from the tip of rostrum to the posterior margin of the caudal rami. Morphological nomenclature follows Huys and Boxshall (1991). The following abbreviations were used in the morphologic description and tables: P1–P6, first to sixth swimming legs; EXP, exopod; ENP, endopod. Setae or spiniform setae are referred to as setal elements. The type specimens were deposited in the collection held at Museo De Historia Natural Marina De Colombia - MAKURIWA.

## ﻿Results

### ﻿Taxonomy

**Order Harpacticoida G.O. Sars, 1903**

**Family Ameiridae Boeck, 1865**

**Subfamily Ameirinae Boeck, 1865**

#### Genus *Nitokra* Boeck, 1865

##### ﻿ Nitokra puebloviejensis sp. nov.

Taxon classificationAnimaliaHarpacticoidaAmeiridae

5AF16EEF-611F-5A5A-9AD1-421032313815

https://zoobank.org/90D3530F-D17B-48E7-BC9A-90F41ED5C0E5

**Figure 1.**
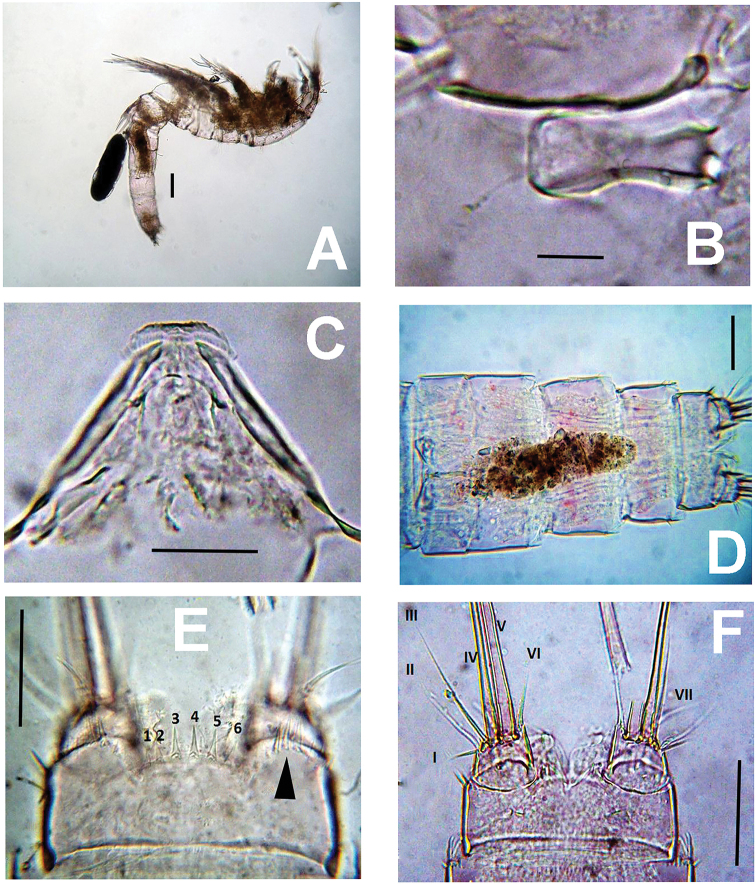
*Nitokrapuebloviejensis* sp. nov. from Puebloviejo, Ciénaga Grande de Santa Marta, Colombia, digital photos. **A** holotype female, habitus, lateral view **B** rostrum, ventral view **C** labrum, ventral view **D** urosomites and caudal rami, ventral view **E** anal somite with anal operculum and spine ornamentation, dorsal view **F** anal somite and caudal rami showing caudal setae I–VII, ventral view. Scale bars: 50 μm (**A, C**); 10 μm (**B**); 20 μm (**E, F**).

**Figure 2.**
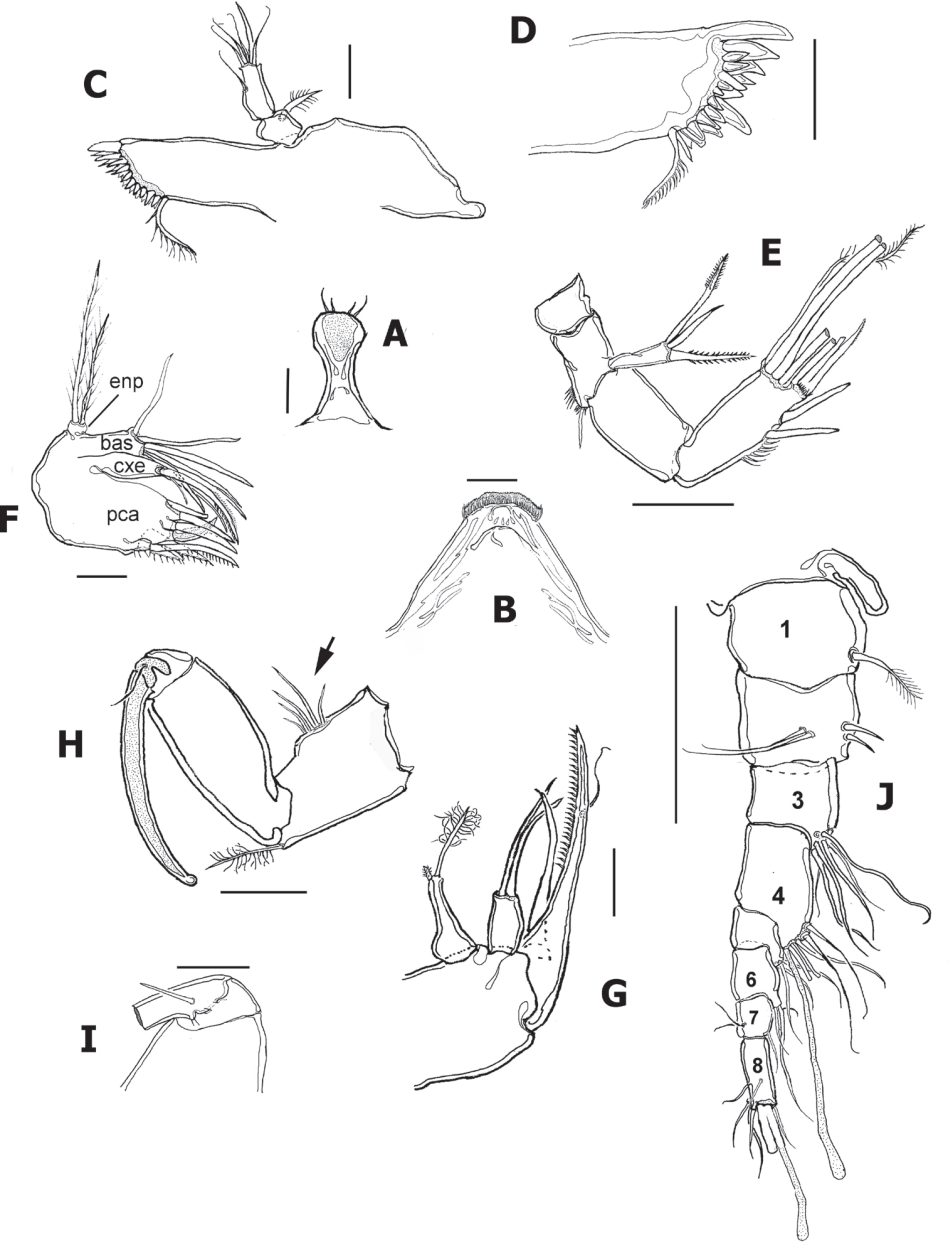
*Nitokrapuebloviejensis* sp. nov. from Puebloviejo, Ciénaga Grande de Santa Marta, Colombia. Adult female holotype. **A** rostrum **B** labrum **C** mandible with palp **D** gnathal blade with teeth and dorsal seta **E** antenna (some setae cut short) **F** maxillule showing armature of lobes **G** maxilla **H** maxilliped showing row of setal elements on syncoxa **I** detail of accessory seta of maxilliped ENP **J** antennule showing segmentation. Scale bars: 15 μm (**A–D, I**); 20 μm (**D–F**); 25 μm (**G, H, J**).

**Figure 3.**
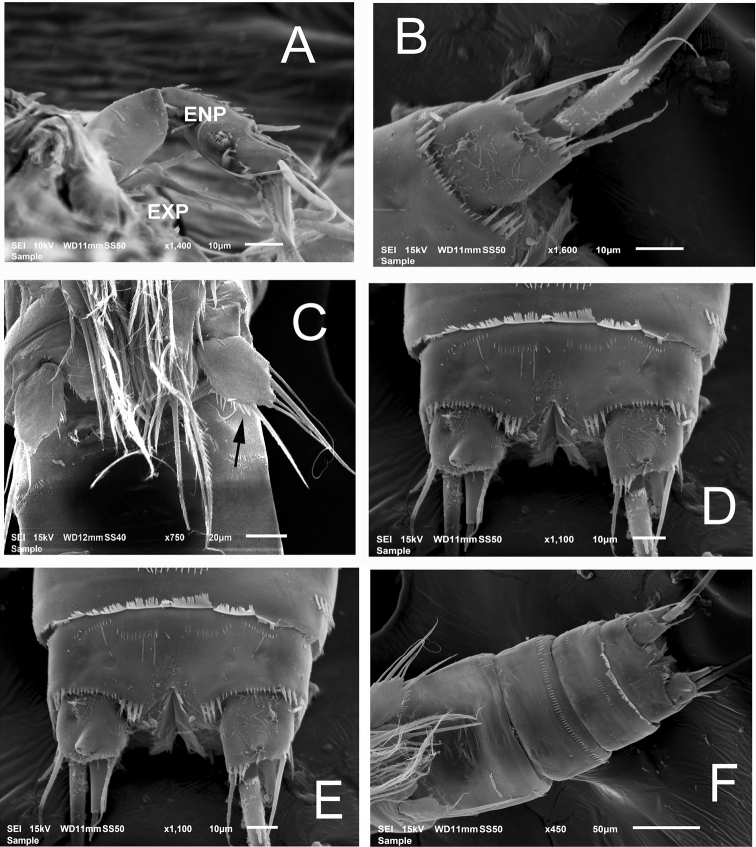
*Nitokrapuebloviejensis* sp. nov. from Puebloviejo, Ciénaga Grande de Santa Marta, Colombia. SEM-prepared adult female. **A** antenna showing EXP and ENP **B** posterior end of anal somite and insertion of caudal rami showing ornamentation, semi-lateral view **C** fifth leg exopods (at arrow), ventral view **D** anal somite and caudal rami, ventral view **E** anal somite, ventral view **F** urosome, ventral view.

**Figure 4.**
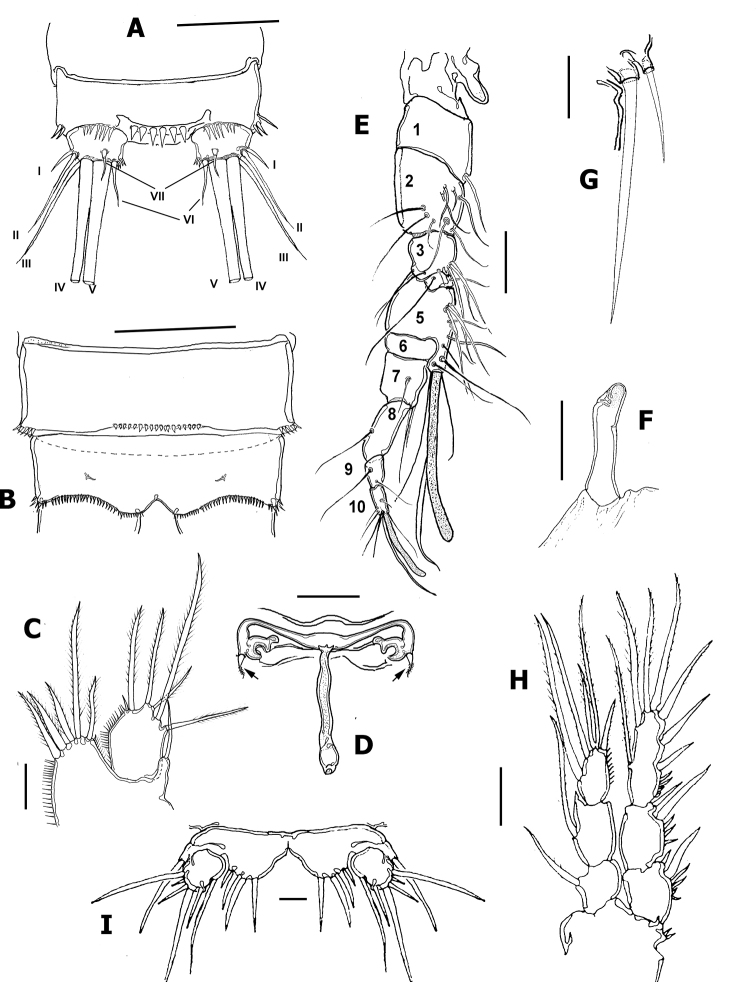
*Nitokrapuebloviejensis* sp. nov. from Puebloviejo, Ciénaga Grande de Santa Marta, Colombia. **A** adult female anal and preanal urosomites, dorsal view showing anal operculum and caudal setae I–VII **B** female anal and preanal urosomites, ventral view **C** female P5 **D** female genital field and P6 with setae (arrowed) **E** male geniculated antennule **F** modified, club-shaped basipodal spine of male P1 **G** male leg 6 **H** male P3, anterior view **I** male P5, ventral view. Scale bars: 25 μm (**A–D, I**); 10 μm (**E–G**); 20 μm (**H**).

**Figure 5.**
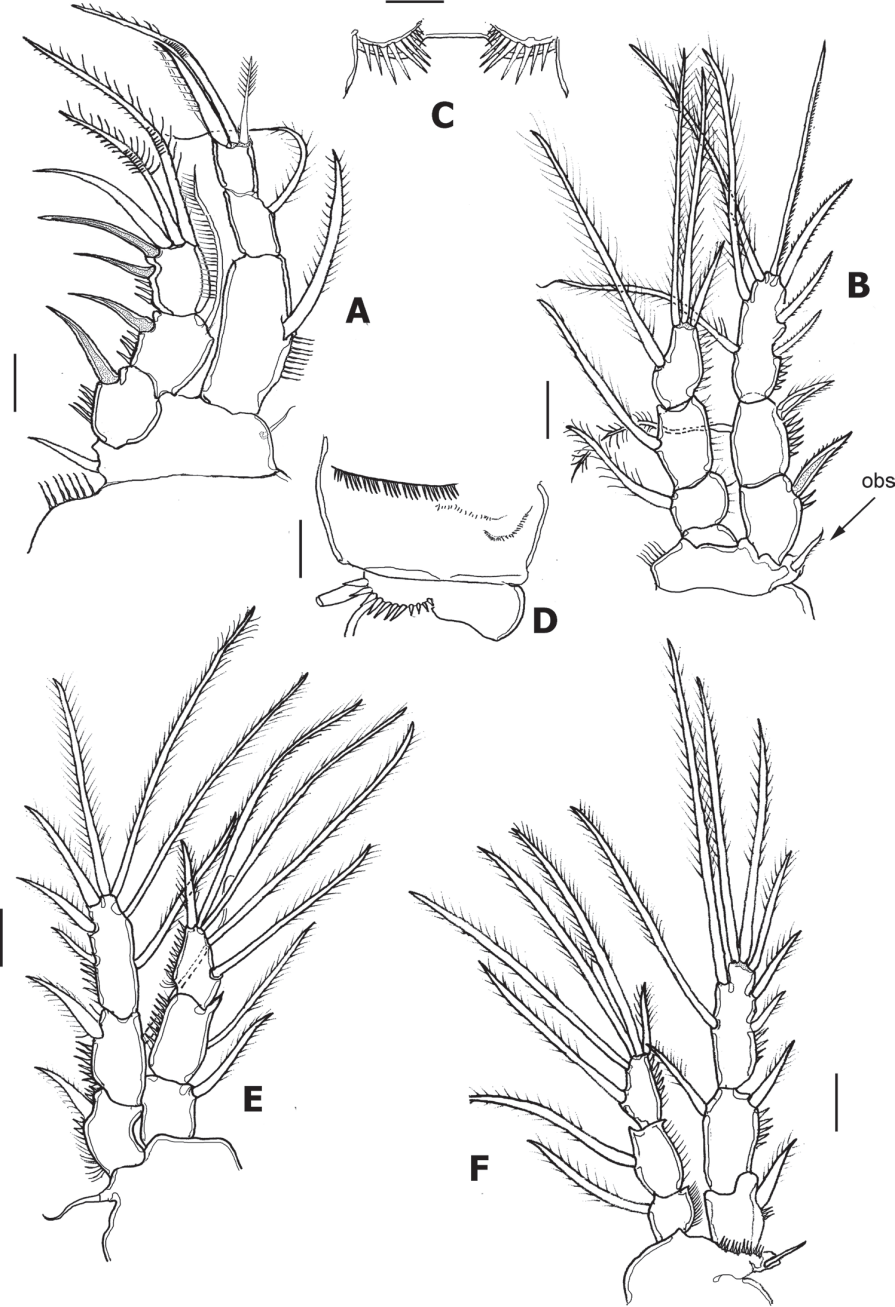
*Nitokrapuebloviejensis* sp. nov. from Puebloviejo, Ciénaga Grande de Santa Marta, Colombia. Adult female holotype **A** leg 1 **B** leg 2 **C** leg 2 intercoxal sclerite **D** leg 2 coxa and basipod ornamentation, anterior view **E** leg3 **F** leg 4. Scale bars: 20 μm (**A, B, D–F**); 10 μm (**C**).

**Figure 6.**
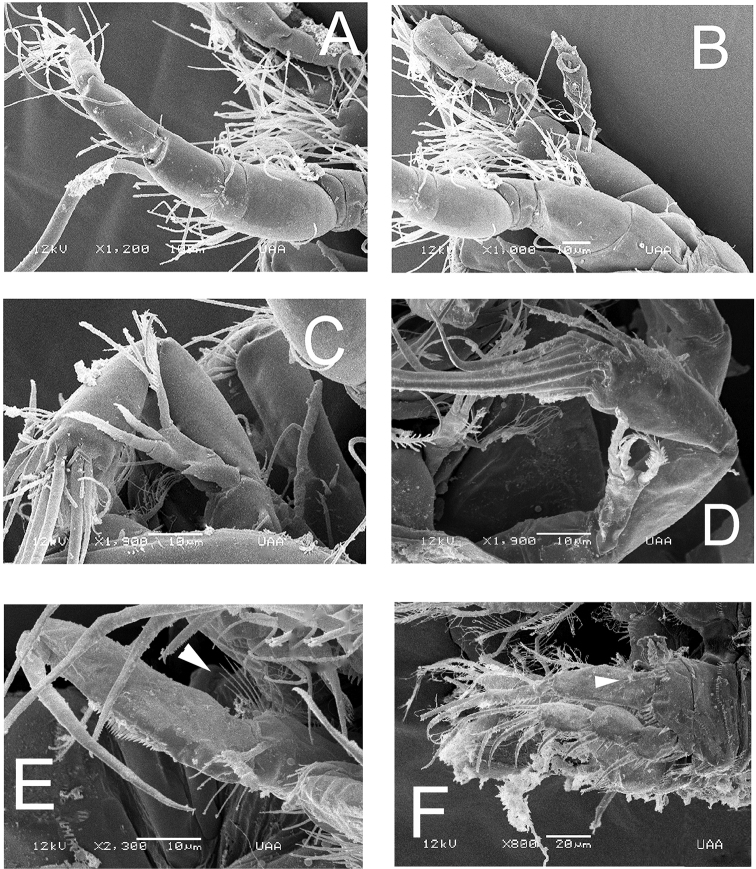
*Nitokrapuebloviejensis* sp. nov. from Puebloviejo, Ciénaga Grande de Santa Marta, Colombia. SEM-prepared adult male individual **A** antennules, semi-lateral view **B** same, showing detail of proximal segments **C** antenna, posterior view **D** same showing exopodal ramus and endopodal segments **E** maxilliped, ventral view showing row of setal elements on syncoxal medial surface (arrowhead) **F** leg 1 showing modified, club-shaped basipodal spine (at arrow).

Figs 1 , 2 , 3 , 4 , 5 , 6

###### Material examined.

Adult female holotype (INV10139), ethanol-preserved, vial, Ciénaga Grande de Santa Marta, Colombia (10°5211.25"N, 74°19'31.64"W), littoral plankton, coll. J.M. Fuentes-Reinés; adult male allotype (INV CRU10140), ethanol-preserved, vial, same sampling data as holotype. Paratypes: four females (INV CRU10141), and three males (INV CRU10142), same sampling data as holotype and allotype.

###### Additional material.

Six adult females, four adult males in first authors’ collection. One female and two male individuals prepared for SEM analysis.

###### Type locality.

Puebloviejo, Ciénaga Grande de Santa Marta, northern Colombia (10°52'11.25"N, 74°19'31.64"W).

###### Etymology.

The new species is named in reference to the type locality of the new species by adding the toponimic suffix in singular. The gender of the species suffix is feminine to match that of the genus.

###### Differential diagnosis.

*Nitokra* with 1 inner seta and 5 setae on P1EXP2 and EXP3, respectively and 455 and 777 elements on P2–P4ENP3 and P2–P4EXP3, respectively, plus 111 inner setae on P2–P4ENP1, respectively. Female rostrum hourglass-shaped, distal segment of mandibular palp with 6 setal elements. P1ENP1 almost reaching distal margin of P1EXP3. Modified, club-shaped inner basipodal seta on leg1. Maxilliped with distinctive group of 5 setae inserted medially on the syncoxa.

###### Description of female.

Body subcylindrical, tapering posteriorly (Fig. 1A), total body length 728–784 μm (average = 768 μm, *n* = 6; holotype length = 784 μm).

Rostrum small, slightly protruding, discernible in dorsal view; roughly hourglass-shaped, with flat tip; rostrum furnished apically with two pairs of short slender sensilla (Figs 1B, 2A). Labrum subtriangular, strong, heavily chitinized, with rugose edge apically (Figs 1C, 2B). Urosome short, thick (Figs 1D, 3D–F), comprising fifth pedigerous somite, genital double-somite and 3 free abdominal somites. Anal somite furnished with row of spinules on proximal ventral surface and along insertion of caudal rami (Figs 1F, 3D–F, 4A). Anal operculum semicircular, ornamented with 5 or 6 spines (Figs 1E, 4A). Caudal ramus short, subquadrate, armed with 7 caudal setae (Figs 1F, 4A), caudal seta I shorter than ramus; seta II about 2.6× as long as I, seta III on distal outer position, slightly longer than seta II. Setae IV and V thick, long, the former being longest; seta VI about 1.6× as long as seta I. Dorsal seta (VII) simple, about as long as caudal ramus, shorter than seta VI (Figs 1F, 4A).

***Antennule*** 8-segmented, tapering distally (Fig. 4E), first segment robust, subquadrate, unornamented, with single seta. Aesthetasc on fourth segment long, reaching beyond distal end of terminal segment. Fourth segment as long as first. Segmental armature as follows (s = seta, ae = aesthetasc): 1(1s),2(4s),3(6s),4(6+1ae).5(3s),6(2),7(4),8(7s+1ae).

***Antenna*** (Figs 2E, 3A). Coxa short, subquadrate, smooth. Basis subrectangular, lacking abexopodal seta, armed with short distal spine and row of minute spinules at spine insertion. First endopodal segment subrectangular, smooth, second endopodal segment longer than first, furnished with subdistal row of spinules on inner margin (Fig. 3A), with 2 lateral inner spines and 6 apical setal elements, outermost 2 basally fused at insertion. EXP 1-segmented, cylindrical, armed with 3 subequal setal elements (Figs 2E, 3A, 6C, D).

***Mandible*** (Fig. 2C, D). Gnathal blade armed with 13 teeth, 6 large, 7 small, 1 long spinule, and long dorsal seta ornamented with short spinules (Fig. 2D). Mandibular palp 2-segmented, basal segment short, subquadrate, with short robust seta (Fig. 2C). Endopodal segment subrectangular, armed with 1 short lateral and 5 apical setae (Fig. 2C).

***Maxillule*** (Fig. 2F). With large praecoxa; precoxal arthrite (pca in Fig. 2F) rectangular, unornamented, armed with group of 4 apical and 2 subapical spiniform elements. Coxal endite shorter than precoxal arthrite, armed with 1 curved serrate spiniform element and 2 smooth setae (cxe in Fig. 2F). Basis (bas in Fig. 2F) shorter than coxal endite, seemingly with five subequal apical and subapical setae; exopod reduced, represented by 1 seta; endopod (enp in Fig. 2F) 1-segmented, armed with 2 subequal plumose setae inserted apically (Fig. 2F).

***Maxilla*** (Fig. 2G). Syncoxa unornamented, with 2 endites, proximalmost armed with short spiniform element and slender modified seta furnished with distal tuft of setules; second endite with 2 slender apical setae. Allobasis produced into strong serrate claw with short, curved adjacent spiniform element and slender seta. ENP 1-segmented, armed with 2 setae.

***Maxilliped*** (Fig. 2H, I). Subchelate. Syncoxa with single setulated seta on inner distal corner and distinctive group of 4 or 5 short slender elements inserted medially on the outer margin of the syncoxa, as in male (Fig. 2H, arrowhead in Fig. 6E). ENP represented by long, slender claw with short accessory seta (Fig. 2I).

P1 (Fig. 5A). Intercoxal sclerite smooth. Coxa with transverse row of spinules proximally and spinules row distally. Basis with spinules bordering insertion of exopodal and endopodal rami, inner basipodal spine short, reaching proximal 1/3 of length of first ENP segment. Outer basipodal spine short. EXP and ENP 3-segmented. Exopodal ramus shorter than endopod, reaching about the margin end of second exopodal segment. EXP1lacking inner setae, EXP2 with inner seta; EXP3 with 3 outer spines and 2 geniculate apical setae. ENP 3-segmented; ENP 2 with inner seta, ENP1 subrectangular. ENP3 with 1 apical spiniform seta, 1 geniculate apical seta, and short plumose inner seta.

P2 (Fig. 5B–D). Intercoxal sclerite with transverse rows of long spinules (Fig. 6C). Coxa with transverse row of long spinules plus 2 rows of minute spinules (Fig. 6D). Outer basipodal spine short (“obs” in Fig. 6B). EXP and ENP 3-segmented. EXP1 lacking inner seta, EXP2 with long, slender inner seta, EXP3 with 3 outer spines, 1 apical and 3 inner setae. ENP shorter than EXP, reaching slightly beyond halfway of EXP3. ENP-3 with 2 inner setae (Fig. 5B).

P3 (Fig. 5E). Intercoxal sclerite smooth. Coxa and basis as in P2. EXP and ENP 3-segmented. ENP slightly shorter than EXP. ENP as in P2 except for additional inner plumose seta on ENP3.

P4 (Fig. 5F). Intercoxal sclerite, coxa and basis as in P2 and P3. EXP and ENP 3-segmented. ENP shorter than EXP, barely reaching 1/3 of EXP3. EXP as in P3 except for thinner outer spines on EXP3. ENP as in P3.

P5 (Fig. 3C at arrow, 4C). EXP and baseoendopod not fused, baseoendopod subtriangular, reaching EXP midlength, segment bearing 5 setae, apical being longest. EXP subquadrate, with spinules row along inner margin; EXP armed with 5 unequally long setae (Fig. 4C).

P6 (Fig. 4D). Represented by narrow transverse plate with subdistal lobe-like processes marked by a rounded notch. Plate bearing a small seta on each side (Fig. 4D at arrows).

Armature formula of female P1–P5 as follows:

**Table:** 

	Exopod	Endopod
P1	I-0; I-1; III,2,0	0-1;0-1;I,I,2,0
P2	I-0; I-1;III,2,2	0-1;0-1; I,2,1
P3	I-0; I-1;III,2,2	0-1;0-1; I,2,2
P4	I-0;I-1;III,2,2	0-1;0-1;I,2,2
P5	5	5

**Male.** Smaller than female, total body length 578–588 μm (average length = 578 μm, *n* = 3; allotype specimen length = 588 μm).

Anal operculum, rostrum, antennae (Fig. 6C, D), and mouthparts as in female. Sexual dimorphism expressed in the antennule, urosome, P1basis, distal inner seta of P3ENP3, P5, and P6.

***Antennule*** (Fig. 6A, B) haplocer, 10-segmented; armature formula difficult to discern, purportedly as follows: 1(1s), 2(9s), 3(6s), 4(2s), 5(9+ae), 6(1s),7(3s), 8(1s),9(2s),10(7+ae).

***Antenna*** (Fig. 6C, D), maxilliped (Fig. 6E), mouthparts, and P1–P4 as in female. Ventral ornamentation of urosomites as in female except urosomite 2. Posterior margins of urosomites with row of small spinules.

P1 basis and P3ENP3. P1 basis with modified, club-shaped inner spine (Fig. 4F, arrowheads in Fig. 6F). P3ENP3 with distal inner spine thinner than in female.

P5 (Fig. 4I). EXP and baseoendopod separated. The former subquadrate, with 5 or 6 setae, Baseoendopod reaching about proximal 1/3 of EXP, armed with 4 elements.

P6. With 2 unequal setae, inner one about 3× as long as outer seta. Caudal rami as in female.

###### Variability.

One male with 3 setal elements (instead of 4) on P5ENP. Another male was observed to possess 6 setae instead of 5 on P5EXP.

###### Habitat.

The new species is known only from the type locality, Puebloviejo, Ciénaga Grande de Santa Marta (northern Colombia). The site where it was collected is a shallow mangrove area, 0.7 m deep, with water temperature 26–31 °C; local salinity was 15–20 PSU, and pH values was 7.5–8.1.

## ﻿Discussion

There are 23 species of the genus reported from the Americas, as follows: *N.typicatypica* Boeck, 1865, *N.spinipesspinipes* Boeck, 1865, *N.lacustrislacustris* (Schmankevitch, 1875), *N.hibernicahibernica* (Brady, 1880), *N.lacustrissinoi* Marcus & Por, 1961, *N.pusilla* Sars, 1911, *N.bdellurae* (Liddell, 1912), *N.affinisaffinis* Gurney, 1927, *N.affinisaffiniscolombiensis* Fuentes-Reinés & Suárez-Morales, 2014, *N.dubia* G. O. Sars, 1927, *N.minorminor* Willey, 1930, *N.chelifer* Wilson, 1932, *N.hyperidis* Jakobi, 1956, *N.fragilispaulistana* Jakobi, 1956, *N.spinipesarmata* Lang, 1965, *N.affiniscalifornica* Lang, 1965, *N.lacustriscolombiana* Reid, 1988, *N.sphaeromata* Bowman, 1988, *N.galapagoensis* Mielke, 1993, *N.bisetosa* Mielke, 1993, *N.evergladensis*Bruno et al., 2002, *N.taylori* Gómez, Carrasco & Morales-Serna, 2012, and *N.lacustrisrichardi*Karanovic et al., 2015 (Willey 1930; Lang 1948; Humes 1953; Jakobi 1956; Reid 1987, 1988; Mielke 1993; Suárez-Morales et al. 1996, 2006, 2009; Suárez-Morales and Gasca 1998; Bruno et al. 2002; Reid and Williamson 2010; Fuentes-Reinés and Zoppi de Roa 2013; Fuentes-Reinés and Suárez-Morales 2014a).

In a partial revision of *Nitokra*, Gómez et al. (2012) divided the genus into three morphological groups based on the combination of the armature formula of the P1EXP2 and 3. Species in the first group carry one inner seta and four elements on P1EXP2 and EXP3, respectively. The second group is distinguished by the absence of an inner seta on P1EXP2 but bears five setae on P1EXP3. The third, most diverse group contains species bearing one inner seta and five setae on P1EXP2 and EXP3, respectively (as in Fig. 5A). Up to 13 species and subspecies have been assigned to this group, whose members also share a pattern of 4,5,5 and 7,7,7 setal elements on P2–P4ENP3 and P2–P4EXP3, respectively, plus 1,1,1 inner setae on P2–P4ENP1, respectively. The group includes: *N.spinipes* Boeck, 1864, *N.fragilisfragilis* Sars, 1905, *N.fragilispaulistana* Jakobi, 1956, *N.spinipesorientalis* Sewell, 1924, *N.pietschmanni* Chappuis, 1933, *N.australis* Soyer, 1974, *N.intermedia* Pesce, 1983, *N.laingensis* Fiers, 1986, *N.husmanni* Kunz, 1976, *N.koreanus* Chang, 2007, *N.taylori* Gómez, Carrasco & Morales-Serna, 2012, *N.vietnamensis* Tran & Chang, 2012, and now *N.puebloviejensis* sp. nov. Within this group, the new species most closely resembles *N.vietnamensis* because they share of several characters including the number of elements on the maxillule coxa, the setation pattern on female P5EXP and P1–P4, relative length of female P5 setophore, number of spines on anal operculum, and relative length of P5ENP inner seta. These two species can be distinguished by the following characters: 1) the distal segment of the mandibular palp has 6 elements in *N.puebloviejensis* (Fig. 2C) vs.\ only 4 in *N.vietnamensis* (Tran and Chang 2012: Fig. 4E), 2) in the new species, *N.puebloviejensis*, the P1ENP1 almost reaches the distal margin of P1EXP3, whereas in *N.vietnamensis* the P1ENP1 is relatively shorter, barely reaching halflength of P1EXP2 (Tran and Chang 2012), 3) in *N.puebloviejensis* sp. nov. both the P2ENP and P3ENP reach about half of P2EXP3 and P3EXP3, respectively (Fig. 5B, E), whereas in *N.vietnamensis* these rami are relatively shorter, barely reaching the proximal 1/3 of P2EXP3 and P3EXP3, respectively (Tran and Chang 2012), 4) the female P5EXP is subquadrate, robust in *N.puebloviejensis* (Fig. 4C) vs clearly narrower and elongate in *N.vietnamensis* (Tran and Chang 2012), 5) the male P5EXP and P5ENP are armed with 5 or 6 and 4 elements, respectively, vs 3 and 6 elements, respectively, in *N.vietnamensis* (Tran and Chang 2012), 6) the male antennule is 10-segmented in *N.puebloviejensis* (Fig. 6A, B) vs 8-segmented in *N.vietnamensis* (Tran and Chang 2012).

Furthermore, *N.puebloviejensis* can be confused with *N.taylori*, another congener known from the Colombian Caribbean, but these two species can be distinguished by the following characters: 1) maxillule coxa with 3 setal elements in *N.puebloviejensis* (Fig. 2F) vs 2 in *N.taylori* (Gómez et al. 2012: Fig. 4C), 2) P1ENP1 almost reaches the distal margin of P1EXP3 (Fig. 5A) vs relatively shorter, reaching only halflength of P1EXP3 in *N.taylori* (Gómez et al. 2012: Fig. 4D; Fuentes-Reinés and Suárez-Morales 2014a: fig 6D), 3) P2ENP and P3ENP reach half the length of P2 EXP3 and P3EXP3, respectively (Fig. 5B, E) vs barely reach the proximal 1/3 of P2EXP3 and P3EXP3, respectively in *N.taylori* (Gómez et al. 2012: figs 5B, 6C; Fuentes-Reinés and Suárez-Morales 2014a: Fig. 6E, F), 4) intercoxal sclerite of P3 is smooth vs with 2 conspicuous spinule rows in *N.taylori* (Gómez et al. 2012, fig 6C), 5) the female P5 EXP is armed with 5 setae in the new species (Figs 3C, 4C) vs 6 such setae in *N.taylori* (Gómez et al. 2012, Fig. 7B, 9C, 10C, Fuentes-Reinés & Suárez-Morales, 2014a, fig 7B, 8G), 6) the female P6 bears a short seta in *N.puebloviejensis* (Fig. 4D) vs 2 slender setae in *N.taylori* (Gómez et al. 2012, Fig. 3D, Fuentes-Reinés & Suárez-Morales, 2014a, fig 8H), 7) female anal operculum with 5 or 6 spines in *N.puebloviejensis* (Fig. 4A) vs 3–5 in *N.taylori* (Gómez et al. 2012: Fig. 3A; Fuentes-Reinés and Suárez-Morales 2014a: Fig. 7C–E), 8) male P5ENP with 4 setae (Fig. 4I) vs 3 in *N.taylori* (Gómez et al. 2012: figs 9C, 10C; Fuentes-Reinés and Suárez-Morales 2014a: fig 8G), 9) male antennule 10-segmented (Fig. 6A, B) vs 9-segmented in *N.taylori* (Gómez et al. 2012; Fuentes-Reinés and Suárez-Morales 2014a), and 10) dorsal caudal seta VII simple (Fig. 4A) vs articulated in *N.taylori* (Gómez et al. 2012). Overall, the new species can be readily distinguished from its known congeners by the maxilliped armature, with a group of five elements on the medial surface of the syncoxa (Fig. 2H, arrowhead in Fig. 6E).

The diversity of the ameirid harpacticoid fauna could be underestimated and deserves further study in the Caribbean region.

### ﻿Key to species of *Nitokra* reported in the Americas

**Table:** 

1	P4EXP3 with 6 elements	**2**
1a	P4EXP3 with 7 elements	**3**
1b	P4EXP3 with 8 elements	**21**
2	P2 and P3EXP3 with 7 and 7 elements, respectively	***N.bisetosa* Mielke, 1993**
–	P2 and P3EXP3 with 6 and 5 elements, respectively	***N.lacustrisrichardi* (Karanovic, 2015)**
3	P5 female endopodite and exopodite with 6 and 5 elements. respectively	**4**
–	P5 female endopodite and exopodite with 5 and 5 elements, respectively	**13**
4	P4ENP1 with inner seta	**5**
–	P4ENP1 without inner seta	**8**
5	P2EXP3 with seven elements	**6**
–	P2EXP3 with six elements	**7**
6	P1EXP3 almost reaching P1ENP3, anal operculum with 3 or 4 spines	***N.taylori* Gómez, Carrasco & Morales-Serna, 2012**
–	P1EXP3 reaching the end margin of P1ENP2, anal operculum without spines	***N.bdellurae* (Liddell, 1912)**
7	P1ENP1 reaching the half of P1EXP3, anal operculum about 15 spines, P5EXP male with 5 elements	***N.chelifer* Wilson, 1932**
–	P1ENP1 overpassing P1EXP3, anal operculum with 9 spines, P5EXP male with 4 elements	***N.typicatypica* Boeck, 1865**
8	P1ENP1 overpassing P1EXP3	**9**
–	P1ENP1 not reaching beyond P1EXP3	**10**
9	P2 and P3ENP3 with 4 and 5 elements, respectively; P3EXP3 with 6 elements; anal operculum lacking spines	***N.pusilla* Sars, 1911**
–	P2 and P3ENP3 with 2 and 3 elements, respectively; P3EXP3 with 5 elements; anal operculum with about 10 spines	***N.hibernicahibernica* (Brady, 1880)**
10	P1ENP1 as long as EXP1 and EXP2 combined	**11**
10a	P1ENP1 reaching 1/3 or ½ of P1EXP3	***N.lacustrissinoi* Marcus & Por, 1961**
10b	P1ENP1 reaching ½ of P1EXP2	**12**
11	Anal operculum with 7 spines, baseoendopodite of P5 female almost reaching distal end of EXP	***N.evergladensis* Bruno & Reid, 2002**
–	Anal operculum with about 10 spines, P5 baseoendopodite reaching middle of EXP	***N.dubia* Sars, 1927**
12	With a spinulate medial surface of the caudal rami and caudal seta II about 2.2 as long as seta I	***N.lacustriscolombiana* Reid, 1988**
–	Spinulate medial surface absent in the caudal rami and caudal seta II less 2 times as long as seta I	***N.lacustrislacustris* (Schmankevitch, 1875)**
13	P4ENP3 with 4 elements	***N.hyperidis* Jakobi, 1956**
–	P4ENP3 with 5 elements	**14**
14	P2ENP3 with 4 elements, P4ENP2 with 1 element	**15**
–	P2ENP3 with 3 elements, P4ENP2 unarmed	***N.galapagoensis* Mielke, 1997**
15	P2ENP1 and P4ENP1 without inner set	***N.minorminor* Willey, 1930**
–	P2ENP1 and P4ENP1 with inner seta	**16**
16	P1ENP1 reaching the margin end of P1EXP2	***N.fragilispaulistana* Jakobi, 1956**
–	P1ENP1 longer than P1EXP2	**17**
17	P1EXP3 reaching the insertion point of inner seta of ENP1	***N.sphaeromata* Bowman, 1988**
–	P1EXP3 going beyond the insertion point of inner seta ENP1	**18**
18	Anal operculum with 6 spines; female and male P6 with 1 and 2 setal elements, respectively; male P5EXP with 5 elements; maxilliped with 4 or 5 setal elements on the syncoxa medial surface	***N.puebloviejensis* sp. nov.**
–	Characters not as above	**19**
19	Anal operculum with 10–15 spines, female and male P6 with 2 and 3 setal elements, respectively; male P5EXP with 6 elements; maxilliped lacking medial setae on the syncoxa	**20**
20	Anal operculum with 10–12 spines, posterior margin of genital double somite with row of long lateral spinules	***N.spinipesarmata* Lang, 1965**
–	Anal operculum with 12–15 spines, posterior part of double genital somite with short row of lateral spinules	***N.spinipesspinipes* Boeck, 1865**
21	Rostrum with long projection	***N.affiniscolombiensis* Fuentes-Reinés & Suárez-Morales, 2014**
–	Rostrum without long projection	**23**
23	P1ENP1 longer than exopodite, posterior edge of antepenultimate somite spinulose dorsal to ventrolateral	***N.affinisaffinis* Gurney, 1927**
–	P1ENP1 as long as exopodite, posterior edge of antepenultimate somite encircled by spinules	***N.affiniscalifornica* Lang, 1965**

## Supplementary Material

XML Treatment for Nitokra puebloviejensis
